# Interdisciplinary development of an overall process concept from glucose to 4,5-dimethyl-1,3-dioxolane via 2,3-butanediol

**DOI:** 10.1038/s42004-023-01052-8

**Published:** 2023-11-16

**Authors:** William Graf von Westarp, Jan Wiesenthal, Jan-Dirk Spöring, Hendrik G. Mengers, Marvin Kasterke, Hans-Jürgen Koß, Lars M. Blank, Dörte Rother, Jürgen Klankermayer, Andreas Jupke

**Affiliations:** 1 Fluid Process Engineering (AVT.FVT), RWTH Aachen University, Forckenbeckstraße 51, 52074 Aachen, Germany; 2 Institute of Technical and Macromolecular Chemistry (ITMC), RWTH Aachen University, Worringerweg 2, 52074 Aachen, Germany; 3 Institute for Bio- and Geosciences Plant Sciences (IBG-1), Forschungszentrum Jülich GmbH, Wilhelm-Johnen-Straße, 52428 Jülich, Germany; 4 Aachen Biology and Biotechnology (ABBt), RWTH Aachen University, Worringerweg 3, 52074 Aachen, Germany; 5 Institute of Applied Microbiology (iAMB), Aachen Biology and Biotechnology (ABBt), RWTH Aachen University, Worringerweg 1, 52074 Aachen, Germany; 6 Institute of Technical Thermodynamics (LTT), RWTH Aachen University, Schinkelstraße 8, 52062 Aachen, Germany

**Keywords:** Chemical engineering, Biocatalysis, Sustainability, Process chemistry

## Abstract

To reduce carbon dioxide emissions, carbon-neutral fuels have recently gained renewed attention. Here we show the development and evaluation of process routes for the production of such a fuel, the cyclic acetal 4,5-dimethyl-1,3-dioxolane, from glucose via 2,3-butanediol. The selected process routes are based on the sequential use of microbes, enzymes and chemo-catalysts in order to exploit the full potential of the different catalyst systems through a tailor-made combination. The catalysts (microbes, enzymes, chemo-catalysts) and the reaction medium selected for each conversion step are key factors in the development of the respective production methods. The production of the intermediate 2,3-butanediol by combined microbial and enzyme catalysis is compared to the conventional microbial route from glucose in terms of specific energy demand and overall yield, with the conventional route remaining more efficient. In order to be competitive with current 2,3-butanediol production, the key performance indicator, enzyme stability to high aldehyde concentrations, needs to be increased. The target value for the enzyme stability is an acetaldehyde concentration of 600 mM, which is higher than the current maximum concentration (200 mM) by a factor of three.

## Abstract (web summary)

The bio-hybrid fuel 4,5-dimethyl-1,3-dioxolane can be produced from glucose *via* 2,3-butanediol (2,3-BDO), however, the separation of microbially produced 2,3-BDO remains costly. Here, the authors develop an alternative process route to 4,5-dimethyl-1,3-dioxolane *via* the enzymatic production of 2,3-BDO.

## Introduction

The reduction of carbon dioxide (CO_2_) emissions is a global challenge. Since mobility and transportation are crucial for our society, so-called carbon-neutral fuels derived from renewable resources have recently gained renewed attention^1^. In an ongoing effort to develop carbon-neutral fuels, the cluster of excellence “The Fuel Science Center” established the novel concept of “bio-hybrid fuels”^2,3^. The idea is to identify fuel candidates with high energy density and laminar burning velocity that can be synthesized from renewable biomass or CO_2_ as feedstock in combination with molecular hydrogen (H_2_)^4^. In particular, promising fuel candidates were identified to be part of the class of cyclic acetals^5,6^.

**Fig. 1:**
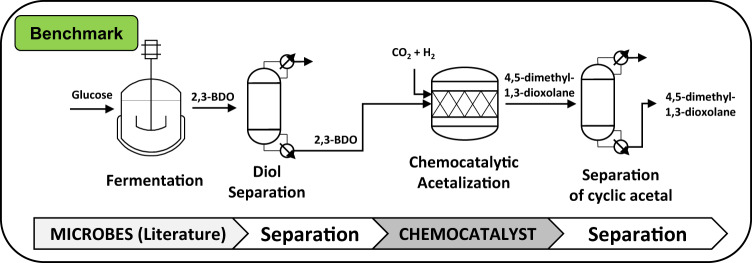
Benchmark process for the production of 4,5-dimethyl-1,3-dioxolane. Benchmark process for the production of 4,5-dimethyl-1,3-dioxolane, consisting of the glucose-based fermentation for the production of 2,3-butanediol (2,3-BDO) and the chemo-catalytical acetalization with addition of CO_2_ and H_2_ to obtain 4,5-dimethyl-1,3-dioxolane as well as the corresponding separation sequences.

In this study, overall process concepts for the production of the cyclic acetal 4,5-dimethyl-1,3-dioxolane via the intermediate 2,3-butanediol (2,3-BDO) were developed. The cyclic acetal can be accessed via chemo-catalytic conversion of biomass-derived 2,3-BDO^7^. In order to assess process concepts to produce 4,5-dimethyl-1,3-dioxolane, a benchmark process for the production of 4,5-dimethyl-1,3-dioxolane was defined:

The typical route for the synthesis of the biomass-derived intermediate 2,3-BDO is the fermentative production from glucose, which has been studied extensively in the last decades^8^. The benchmark for the separation of 2,3-BDO from the aqueous fermentation broth is distillation^9,10^. To produce 4,5-dimethyl-1,3-dioxolane, the purified 2,3-BDO, CO_2_, and H_2_ are inserted to a pressurized reactor where the ruthenium transition metal complex catalyzes the formation of the cyclic acetal^11^. Consecutively, the synthesized 4,5-dimethyl-1,3-dioxolane is separated (see Fig. 1).

While the production and separation of 4,5-dimethyl-1,3-dioxolane is not investigated so far, the main cost factor in the production of 2,3-BDO was identified to be its separation^12^. Due to the high boiling point of 2,3-BDO of 180 °C, large amounts of water, which is the low boiling compound, need to be evaporated in distillation, making the separation very energy intensive. Numerous conceptual studies were performed to address this challenging separation task^13,14^. However, the most practical approach remains the inefficient, but easy to operate distillation of the aqueous solvent^15,16^.

To enable a more efficient 2,3-BDO production, a promising solution is the utilization of a non-conventional micro aqueous reaction system (MARS) for synthesis^17,18^. Here, the aqueous solvent is replaced by organic solvents. The separation of high boiling diols from MARS instead of aqueous solvent can be favorable, due to the lowered enthalpy of evaporation of the organic reaction solvent^19,20^. In previous studies, the production of vicinal diols via enzymatic conversion in MARS has been demonstrated^21,22^. A synthetic enzymatic cascade consisting of a ligation step and a reduction step was utilized for the production of diols, but not 2,3-BDO^19^. In contrast to glucose-based fermentative strategies for the production of 2,3-BDO, the novel enzymatic pathway requires acetaldehyde as educt for the conversion to 2,3-BDO^23^.

**Fig. 2:**
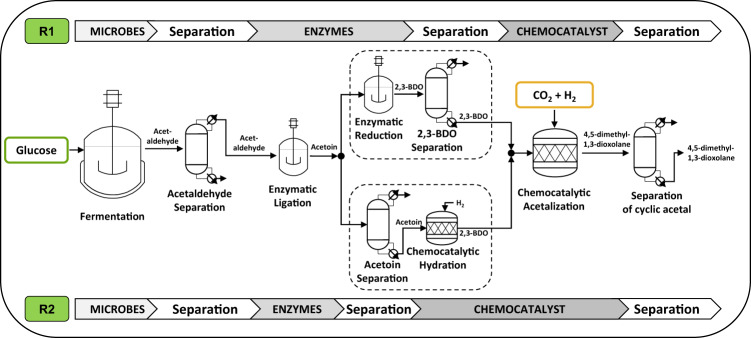
Process routes for the production of 4,5-dimethyl-1,3-dioxolane. Alternative process routes (**R1** & **R2**) for the production of 4,5-dimethyl-1,3-dioxolane from glucose. Acetaldehyde is produced by microbes from glucose and separated consecutively. The obtained acetaldehyde is converted to acetoin in an enzymatic ligation step using an ThDP-dependent enzyme (formulated as whole lyophilized cell). In **R1** acetoin is further converted to 2,3-butanediol (2,3-BDO) using an enzymatic reduction step using an alcohol dehydrogenase and separated from its reaction solvent. In **R2** acetoin is separated from its reaction solvent and subsequently hydrogenated chemo-catalytically to 2,3-BDO by addition of H_2_. In both process routes, the 2,3-BDO is inserted in ain add chemo-catalytic acetalization step under addition of CO_2_ and H_2_ to obtain 4,5-dimethyl-1,3-dioxolane. Finally, 4,5-dimethyl-1,3-dioxolane is purified.

Acetaldehyde can be obtained via glucose-based fermentation^24^. The combination of fermentation and enzymatic conversion of acetaldehyde to 2,3-BDO leads to an alternative process route (**R1**) compared to the benchmark process for 2,3-BDO production (see Fig. 2). The fermentation requires in situ separation of acetaldehyde due to its toxic effects on the microorganisms^25^. For the volatile acetaldehyde, an efficient separation via gas stripping and absorption has been proposed^26–28^. The absorbed acetaldehyde can be recovered at high purities via low-energy requiring distillation^25^. A second possible process route is the substitution of the enzymatic reduction with a chemo-catalytic hydrogenation in the presence of H_2_ (**R2**). Therefore, the established ruthenium catalyst enabling the formation of the cyclic acetal can also be used for the reduction step. In contrast to **R1**, where 2,3-BDO is separated after the enzymatic reduction step, **R2** requires the separation of acetoin before the hydration step.

In addition to the reaction routes (**R1** & **R2**), the choice of the reaction solvent for the enzymatic conversion steps (aqueous or MARS) and the consecutive separation strategies result in degrees of freedom. This is only possible by the fact that the enzymatic steps can be carried out in separate steps rather than in one living organism, leading to multiple possible process routes for the production 4,5-dimethyl-1,3-dioxolane by combining bio-catalysis and chemo-catalysis^17,29^.

Here we show the assessment of the potential of the proposed process concepts to produce 4,5-dimethyl-1,3-dioxolane from glucose in an early stage of catalyst development. The specific energy demand of the benchmark process is compared to the specific energy demand of the proposed process concepts. To this end, the relevant process parameters of each (bio-)catalytic reaction step (yield, titers, stoichiometric reaction equations, and possible reaction solvents) are elucidated in the following section. Secondly, suitable separation sequences for each intermediate (acetaldehyde, acetoin, and 2,3-BDO) as well as the final product (4,5-dimethyl-1,3-dioxolane) are developed. Finally, the most efficient process concept is identified and process parameters for further improvement of the overall process efficiency are discussed.

## Results and discussion

### Process parameters of each (bio-)catalytic reaction

In the subsequent section, the relevant process parameters of the three investigated (bio-)catalyst types (microbes, enzymes, and chemo-catalyst) are described. Special focus is given on the reaction conditions (temperature, pressure, and possible reaction solvents) as well as performance parameter of the catalysts, such as yield and titer.

#### Fermentative production of acetaldehyde

**Fig. 3:**
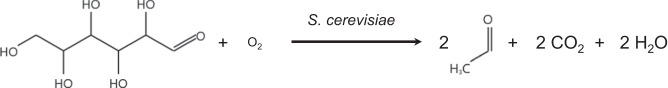
Reaction scheme to acetaldehyde. Fermentative conversion of glucose to acetaldehyde using *S. cerevisiae* at 32 °C and at atmospheric pressure.

The fermentative production of acetaldehyde from glucose (see Fig. 3) via fermentation of engineered yeast was reported in a previous study of our working group^25^.

A genetically modified *S. cerevisiae* was utilized for the production of acetaldehyde in aqueous medium at 32 °C and atmospheric pressure. Acetaldehyde was found to be toxic to the respiratory chain of the yeast. Hence, in situ separation of acetaldehyde is beneficial and due to the reactor’s aeration also inevitable. The aeration is leading to in situ product removal via gas stripping. The acetaldehyde in the off-gas was absorbed in water traps. Overall, 37% of the theoretical yield (0.49 g_acetaldehyde_/g_glucose_) was achieved at laboratory scale (200 mL) and ~75% of the produced acetaldehyde was recovered in the water traps. Based on these findings, the production of acetaldehyde from glucose is integrated into the assessment of a process for the production of 4,5-dimethyl-1,3-dioxolane.

#### Enzymatic synthesis of 2,3-butanediol

**Fig. 4:**
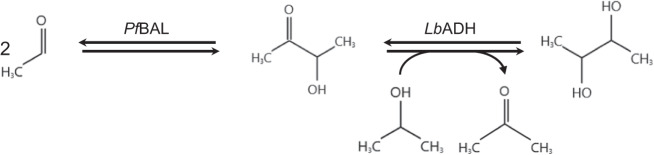
Reaction scheme to 2,3-butanediol. Reaction of acetaldehyde to acetoin and 2,3-butanediol in a two-step enzymatic cascade at 30 °C and atmospheric pressure with integrated substrate-coupled regeneration of the cofactor via isopropanol. The first step is conducted using a lyase (*Pf*BAL) and the second step is performed by a dehydrogenase (*Lb*ADH).

The biocatalytic transformation of acetaldehyde to 2,3-BDO via acetoin in a two-step enzymatic cascade consisting of the benzaldehyde lyase from *Pseudomonas fluorescens* (*Pf*BAL) and alcohol dehydrogenase from *Lactobacillus brevis* (*Lb*ADH) is reported. The first step is a *Pf*BAL catalyzed C-C bond formation in a benzoin type condensation reaction between two acetaldehyde molecules to form acetoin. The acetoin is then reduced using the *Lb*ADH using isopropanol for the regeneration of the cofactor NADH (see Fig. 4).

The enzymes were formulated as lyophilized whole cells, allowing for cost efficient biocatalyst production (details of biocatalyst preparation can be found in Supplementary Methods in Section 1.1 Enzymatic catalysis). The enzymatic reactions were successfully conducted in traditional aqueous reaction solvent as well as in MARS using cyclopentyl methyl ether (CPME). In both cases, the reaction was performed at a temperature of 30 °C and under atmospheric pressure. Details of the reaction conditions and analytics can be found in the Supplementary Methods in Section 1.1.3 and Fig. S1. In the aqueous reaction solvent, the enzymatic ligation of acetaldehyde to acetoin reached a yield of 82.8% and in the consecutive enzymatic reduction of acetoin to 2,3-BDO a yield of 60.3% was achieved. In MARS the enzymatic ligation reached a yield of 59.3% and the yield of the enzymatic reduction was 56.9% (see Fig. S2). The enzymatic ligation in MARS is slightly slower compared to the aqueous reaction solvent and the yield decreased from 82.8 % to 59.3% indicating reaction hindering effects in MARS. Despite these drawbacks in terms of catalytic activity in MARS, in the overall process perspective the MARS offers a more efficient product removal via distillation due to lowered enthalpy of evaporation of the solvent^19^. Therefore, a trade-off between conversion and separation needs to be identified in the process assessment. Additionally, the *Pf*BAL in the enzymatic ligation is inactivated by high concentrations of the reactive acetaldehyde, which has to be addressed as an enzyme specific constraint within process modeling^30^. Hence, low concentrations of acetaldehyde need to be fed, making continuous processing an interesting reaction mode^31^.

#### Chemo-catalytic synthesis of 4,5-dimethyl-1,3-dioxolane

**Fig. 5:**
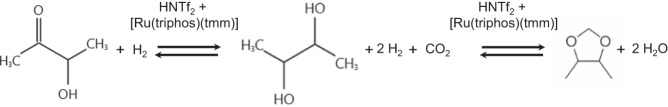
Reaction scheme towards 4,5-dimethyl-1,3-dioxolane. Hydrogenation of acetoin to 2,3-BDO catalyzed by the [Ru(triphos)(tmm)] catalyst at 90 °C and 100 bar. Further, a transformation of 2,3-BDO, CO_2_, and H_2_ to 4,5-dimethyl-1,3-dioxolane catalysed by the same [Ru(triphos)(tmm)] catalyst and a Lewis acid at 90 °C and 80 bar CO_2_/H_2_ in a ratio of 1:3 is presented.

The chemical transformations of acetoin and 2,3-BDO are facilitated by a recently established homogenous [Ru(triphos)(tmm)] catalyst^32,33^. As a co-catalyst the Lewis acid HNTf_2_ is used. Importantly, the solvent used in the enzymatic cascade (CPME) can also be used in the chemical transformation (see Fig. 5)^17^.

The [Ru(triphos)(tmm)] catalyst enabled the hydrogenation of acetoin to 2,3-BDO. It was successfully carried out in CPME, at a temperature of 90 °C, and a pressure of 100 bar H_2_. After 16 h reaction time, full conversion of acetoin to 2,3-BDO was reached. Consecutively, 2,3-BDO was employed together with CO_2_ and H_2_. The reaction was conducted at 90 °C for 18 h at a pressure of 80 bar CO_2_/H_2_ in a ratio of 1:3. For a more detailed description of the experimental conduction and analytics see Supplementary Methods in Section 1.2. Chemocatalytic Conversion. Overall, the final product 4,5-dimethyl-1,3-dioxolane was successfully formed from 2,3-BDO with a yield of 18%. In addition to the effective utilization of CO_2_, the chemo-catalyst can be used adaptively for the direct conversion of acetoin as substrate.

On the one hand, unlike with enzymes, no stereoselective conversion can be achieved using the chemo-catalyst. On the other hand, a very efficient conversion of acetoin can be achieved, offering broader handover opportunities from the previous reaction of the enzymatic cascade.

### Conceptual process design

Conceptual process designs of the proposed process routes are modeled in AspenPlus. Depending on the choice of catalyst and reaction solvent for each conversion step, suitable separation sequences are identified. The design of the envisioned unit operations is performed based on property data from literature as well as experimentally derived equilibrium data. Finally, the modeling results of the process concepts are discussed.

#### Identification of possible separation sequences

Two process routes for the production of 4,5-dimethyl-1,3-dioxolane from glucose via 2,3-BDO are depicted in Fig. 2 (**R1** and **R2**). The routes vary by the choice of the catalyst for the individual conversion steps. For these novel conversion steps and depending on the choice of the reaction solvent, separation strategies need to be developed.

**Fig. 6:**
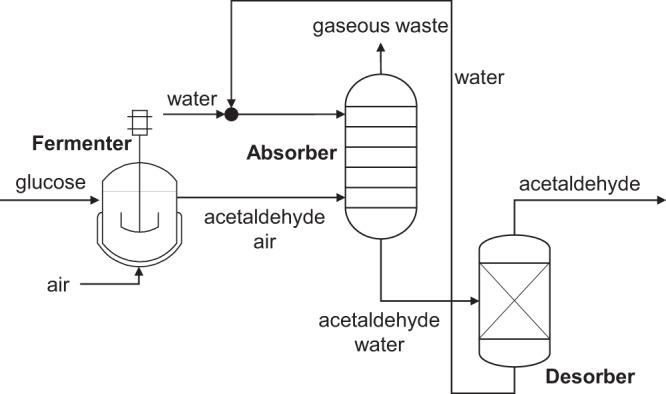
Separation strategy for acetaldeyhde. Acetaldehyde is produced from glucose in a fermentation and is removed from the water phase via the off-gas, and finally captured via absorption in water in both routes, **R1** and **R2**. Consecutively, acetaldehyde is evaporated in a desorber, while the water leaving the column is recycled as absorption solvent into the absorber.

In both reaction routes (**R1** and **R2)**, acetaldehyde is produced from glucose by *S. cerevisiae*. The acetaldehyde is produced in an aqueous medium and a separation via gas stripping and absorption followed by distillation is proposed (see Fig. 5). The absorption based separation of acetaldehyde from the fermentation off-gas was already suggested in literature^25–28^. However, all these studies were conducted in lab scale and the separation of acetaldehyde from the off-gas was rather intended for quantification of the produced acetaldehyde rather than for actual production. We envisioned a counter-current absorber column for the acetaldehyde separation in plant scale to reduce the amount of necessary solvent for absorption. As Mengers et al. suggest, we use water as solvent for absorption, due to its non-toxicity and low cost. The absorption solvent is loaded with acetaldehyde from the fermenter off-gas and the fermenter off-gas leaves the absorber as gaseous waste, mainly consisting of air a small fraction of remaining acetaldehyde. The absorption solvent is recovered in a consecutive desorber via distillation. Water can be recycled as absorption solvent and acetaldehyde as the low boiling compound can be obtained in the head fraction of the distillation column (see Fig. 6).

**Fig. 7:**
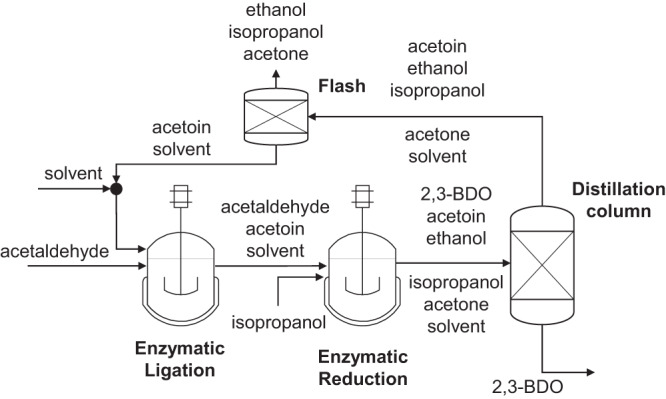
Separation strategy for acetoin or 2,3-butanediol. Acetaldehyde is inserted into an enzymatic cascade where it is converted to 2,3-BDO (**R1**). Thereby, isopropanol serves as co-substrate and acetone results as co-product. The 2,3-BDO is separated from the reaction solution via distillation (prior extraction is possible) while acetoin is recycled, and the co-substrate and co-product are purged. This flowsheet corresponds to the distillation from aqueous solvent (**DIST-AQ**) and the distillation from MARS (**DIST-ORG**).

The purified acetaldehyde is used as educt for the enzymatic ligation, resulting in the intermediate acetoin. In **R1**, the produced acetoin is further processed to 2,3-BDO in an enzymatic reduction by a two-step enzymatic cascade with high stereoselectivity. The enzymatic conversions can be conducted in both, aqueous reaction solvent and MARS. In both cases, isopropanol needs to be added as co-substrate and acetone is produced as co-product. This results in three possible downstream concepts for the separation of 2,3-BDO from the reaction solvent in order to provide the chemo-catalytical step with a solution that is rich in 2,3-BDO and free of side-components. Firstly, the 2,3-BDO can be separated via distillation when formed in an aqueous reaction medium. Thereby, the 2,3-BDO is the high boiling compound and the aqueous solvent, which is the largest amount of the reaction solution, has to be evaporated (distillation from aqueous solvent: **DIST-AQ**). The second option is the distillation of 2,3-BDO from MARS. Once again, major part of the reaction solvent has to be evaporated. However, the evaporation of the organic reaction solvent is less energy intensive in comparison to the aqueous solvent due to the lower enthalpy of evaporation. On the other hand, the conversion yield in organic solvent is lower than in aqueous system leading to a trade-off between these two variants (distillation from MARS: **DIST-ORG**). A schematic presentation of these distillation-based concepts is depicted in Fig. 7. The third possible downstream concept is the extraction of hydrophilic 2,3-BDO from MARS into water with consecutive distillation. Even though the evaporation of water is more energy intensive, significantly less solvent needs to be evaporated, due to a low phase ratio of water to organic reaction solvent (extraction into aqueous solvent: **EXT-DIST**). The ratio can be minimized by applying counter-current multistage extraction.

**Fig. 8:**
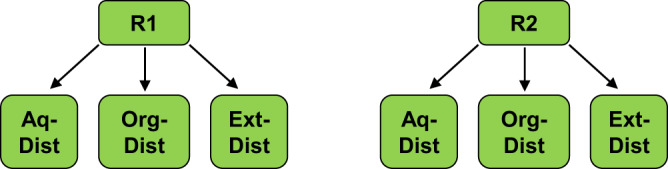
Process variants for the prodution of 4,5-dimethyl-1,3-dioxolane. Six process variants are compared. Two general process routes (R1 and R2) with three downstream concepts (distillation from aqueous solvent: **DIST-AQ**, distillation from MARS: **DIST-ORG**, extraction into aqueous solvent: **EXT-DIST**) are investigated.

In the second process route for the production of 4,5-dimethyl-1,3-dioxolane (**R2**), the enzymatically produced acetoin is separated from the reaction solvent before further processing. The three mentioned separation sequences for 2,3-BDO (distillation from aqueous solvent, distillation from MARS, and combined extraction and distillation) can also be applied for the separation of hydrophilic acetoin from its reaction solvent. This leads to six process variants in total: two process routes (R1 and R2) and three downstream concepts for the intermediates 2,3-BDO or acetoin (**DIST-AQ,** **DIST-ORG,** **EXT-DIST**) as presented in Fig. 8.

Since aqueous reaction solvent and MARS can also be used for acetoin production, the separation task for the separation of acetoin is comparable to 2,3-BDO. After the separation of acetoin via distillation or combined extraction and distillation, acetoin is converted to 2,3-BDO in the chemo-catalytic hydrogenation with addition of H_2_. In contrast to **R1**, the obtained 2,3-BDO can be further processed in the acetalization step without previous separation in **R2**, since the reaction solvent and catalyst are the same for the acetalization as for the chemo-catalytic hydration and no side-components are present^17^.

**Fig. 9:**
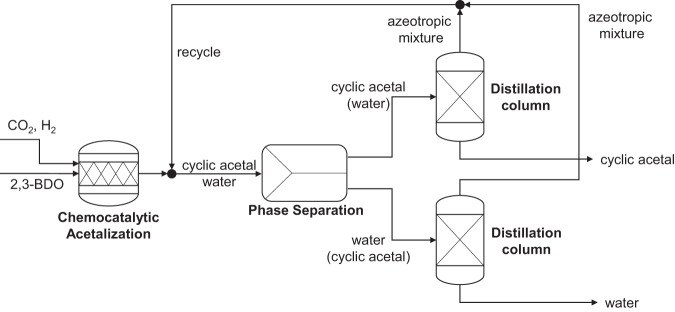
Separation strategy for 4,5-dimethyl-1,3-dioxolane. Produced 2,3-BDO reacts with CO_2_ and H_2_ to 4,5-dimethyl-1,3-dioxolane in the chemo-catalytic acetalization in both process routes (**R1** and **R2**). The reaction mixture is separated via phase separation and distillation. Water and the purified cyclic acetal (4,5-dimethyl-1,3-dioxolane) are obtained.

In all process routes (Benchmark, **R1** and **R2)**, 2,3-BDO is converted with CO_2_ and H_2_ towards the final 4,5-dimethyl-1,3-dioxolane, with water as co-product. The separation of the produced 4,5-dimethyl-1,3-dioxolane is conducted via phase separation and consecutive distillation (see Fig. 9). The mixture of water and 4,5-dimethyl-1,3-dioxolane forms an aqueous and organic rich phase. The final purification of both phases to obtain 4,5-dimethyl-1,3-dioxolane and water is conducted via distillation. In both columns, the bottom stream corresponds to the pure substances, while in the head fraction the corresponding azeotrpoic mixtures consisting of water and 4,5-dimethyl-1,3-dioxolane are obtained in azeotropic composition (approximately 57 mol% 4,5-dimethyl-1,3-dioxolane and 43 mol% water). The azeotropic mixture is recycled to minimize losses of the desired 4,5-dimethyl-1,3-dioxolane.

#### Design of the separation steps

The unit operations for separation in the presented process concepts are absorption, extraction, and distillation. To estimate the costs of the overall process concepts, the separation units need to be designed for the individual application cases occurring in the given process concepts. Since mass transfer in the absorption and extraction is crucial, special focus is on the design of these two unit operations in contrast to well explored distillation^34^.

##### Absorption

Mengers et al. investigated the absorption of acetaldehyde from a diluted gas stream in lab-scale. Water was chosen as a suitable absorption solvent, due to low toxicity, low volatility, and favourable equilibrium for the absorption of acetaldehyde. The equilibrium was described via the Henry coefficient (*H*), which links the molar fraction of a compound in liquid phase (*x*_*i*_) and the partial pressure in the gaseous phase (*p*_*i*_):

$${x}_{i}{{{\cdot }}H}_{i}={p}_{i}$$

The Henry coefficient for acetaldehyde in water was experimentally determined to *H* = 2.59 bar for the binary system and can be used for the design of an absorption column^25^. Therefore, the equilibrium curve of loaded gaseous and liquid streams can be calculated as follows:

$${Y}_{i}=\frac{{H}_{i}\,{{\cdot }}{X}_{i}}{{p}_{{tot}}-{X}_{i}{{\cdot }}({H}_{i}\,-{p}_{{tot}})}$$

where ${Y}_{i}$ is the loading of the gaseous stream, ${X}_{i}$ the loading of the liquid stream, and ${p}_{{tot}}$ is the total pressure^35^. Based on a mass flow of glucose ($\dot{F}=10{kg}/h$) into the fermenter, a theoretical yield of the conversion from glucose to acetaldehyde (${{{{{{\rm{\eta }}}}}}}_{{{{{{\rm{th}}}}}}}=0.49{g}/{g}_{{glucose}}$) and an assumed relative yield of the conversion by the microorganisms (${{{{{{\rm{\eta }}}}}}}_{{MO}}=0.95$), the mass flow $({\dot{m}}_{{acetaldehyde}})$ and molar flow $({\dot{n}}_{{acetaldehyde}})$ of acetaldehyde leaving the fermenter can be determined:

$${\dot{m}}_{{acetaldehyde}}=\dot{F}{{\cdot }}{{{{{{\rm{\eta }}}}}}}_{{MO}}{{\cdot }}{{{{{{\rm{\eta }}}}}}}_{{th}}$$

$${\dot{n}}_{{acetaldehyde}}=\frac{{\dot{m}}_{{acetaldehyde}}}{{M}_{{acetaldehyde}}}$$

where ${M}_{{acetaldehyde}}$ is the molar mass of acetaldehyde (${M}_{{acetaldehyde}}=44.05{g}/{mol}$). The relative yield of the microorganisms of 95% is an assumption, which is based on other biotechnological conversions, e.g., the yeast based production of ethanol and is used in this work to assess the potential of an advanced conversion step^36^. The molar flow of air (${\dot{n}}_{{air}}$) can be derived from its volume flow $({\dot{V}}_{{air}})$, which can be calculated via the productivity of the fermentation ($P=5{g}/L/h$) and a volumetric flow rate (${vvm}=1.5{L}_{{air}}/{L}_{{reactor}}/\!\min$).

$${\dot{V}}_{{air}}={vvm}{{\cdot }}60{{\cdot }}\frac{{\dot{m}}_{{acetaldehyde}}}{P}$$

$${\dot{n}}_{{air}}={\dot{V}}_{{air}}{{\cdot }}\frac{{\rho }_{{air}}}{{M}_{{acetaldehyde}}}$$

Where ${\rho }_{{air}}$ is he density of air (${\rho }_{{air}}=1.204{g}/L$). The productivity and volumetric flow rate are assumptions taken from conventional biotechnological ethanol production processes as mentioned above. Based on the material balances, the entering gas loading $({Y}_{{in}})$ and the leaving gas loading $({Y}_{{out}})$ can be calculated based on a target recovery of 95% $({rec}=0.95)$:

$${Y}_{{in}}=\frac{{\dot{n}}_{{acetaldehyde}}}{{\dot{n}}_{{air}}}$$

$${Y}_{{out}}={Y}_{{in}}{{\cdot }}(1-{rec})$$

**Fig. 10:**
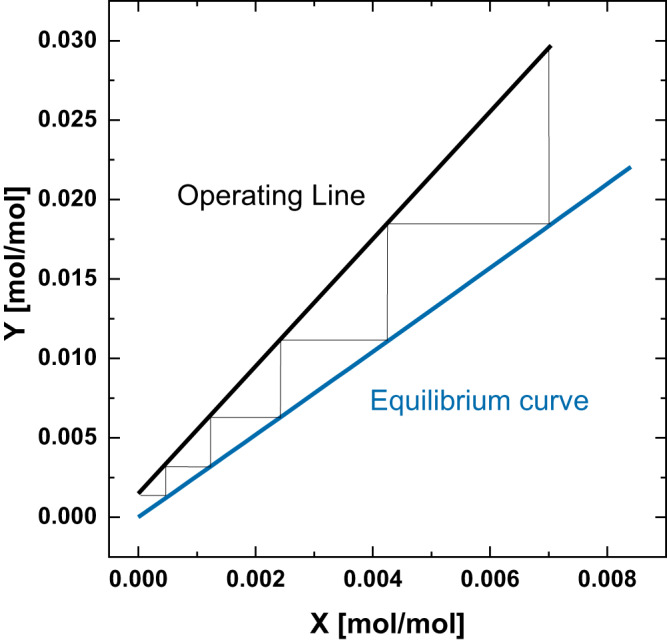
Design of absorption column. Construction of operating line and equilibrium curve for the determination of the number of necessary theoretical separation stages for the recovery of acetaldehyde from the gaseous stream via absorption in water.

Entering gas stream (${Y}_{{in}}$) and leaving solvent stream (${X}_{{out}}$) are assumed to be in equilibrium, when an infinite number of theoretical separation trays is utilized and, hence, a minimum amount of solvent can be used. The minimum amount of necessary solvent ${\dot{L}}_{\min }$ can be calculated as follows:

$${X}_{{out}}=\frac{{Y}_{{in}}{{\cdot }}{p}_{{tot}}}{H+{Y}_{{in}}{{\cdot }}(H-{p}_{{tot}})}$$

$${\dot{L}}_{\min }={\dot{n}}_{{air}}{{\cdot }}\frac{{X}_{{out}}-{X}_{{in}}}{{Y}_{{in}}-{Y}_{{out}}}$$

where the loading of the entering solvent is assumed to be zero $({X}_{{in}}=0).$ Based on the minimum amount of solvent, the practically used amount of solvent is selected to be 1.6 ${\dot{L}}_{\min }$ $({\dot{L}}_{0}=235.33{mol}/\!\min )$^35^. The operating line can be obtained and the number of necessary separation stages can be derived from the operating line and equilibrium curve (${N}_{{th}}=5$) as presented in Fig. 10.

##### Extraction

**Fig. 11:**
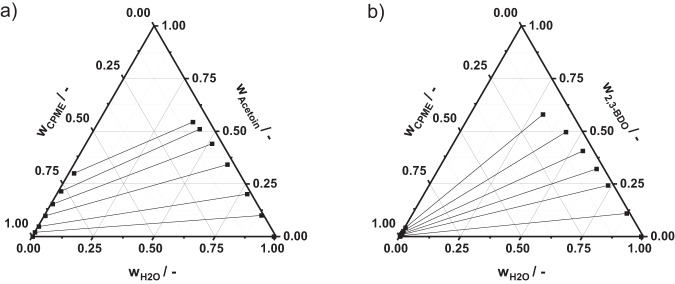
Property data for extraction. Liquid-liquid-equilibrium data obtained from COSMO-RS for the ternary systems consisting of water, CPME, and 2,3-BDO (**a**) and acetoin (**b**).

To design the extraction column for the extraction of acetoin or 2,3-BDO from organic solvent (CPME) to water, the number of theoretical separation stages and the amount of extraction solvent (water) needs to be defined. These two parameters can be approximated by applying the Hunter-Nash-Method, which is based on ternary liquid-liquid-equilibrium (LLE) data^37^. No experimental ternary LLE data is available for the extraction system consisting of water, CPME, and acetoin or 2,3-BDO. Hence, the desired properties were predicted using the conductor-like screening model for real solvation (COSMO-RS) in COSMOthermX19 via activity coefficients. The COSMOtherm internal BP-TZVPD-FINE (Becke-Perdew functional and the triple zeta valence plus polarization function) parametrization was used. The ternary LLE was calculated at 25 °C based on the obtained activity coefficients for the transfer molecules 2,3-BDO (Fig. 11a) and acetoin (Fig. 11b).

Since acetoin as well as 2,3-BDO are highly hydrophilic, in both LLE diagrams the tie-lines indicate an accumulation of the enzymatically produced substances (acetoin or 2,3-BDO) in the aqueous phase. 2,3-BDO is more hydrophilic due to its second hydroxyl-group in contrast to the keto-group of acetoin. Both extraction systems show a wide miscibility gap. The necessary amount of solvent was determined using AspenPlus, based on a desired recovery of 95% and a maximum of ten theoretical separation stages in the counter-current extraction column (${N}_{{th}}=10$). The number of theoretical separation stages was chosen to be lower than ten, due to restrictions of technical equipment^38^. The amount of necessary solvent and the number of theoretical separation stages were investigated via sensitivity analysis in AspenPlus and were consecutively minimized. More details of the proceeding can be found in the Supplementary Methods in Section 1.3 Process Modeling. The proceeding led to a solvent to feed ratio of 0.14 for the extraction of acetoin and to 0.035 for the extraction of 2,3-BDO (see Fig. S5).

##### Phase separation and distillation

**Fig. 12:**
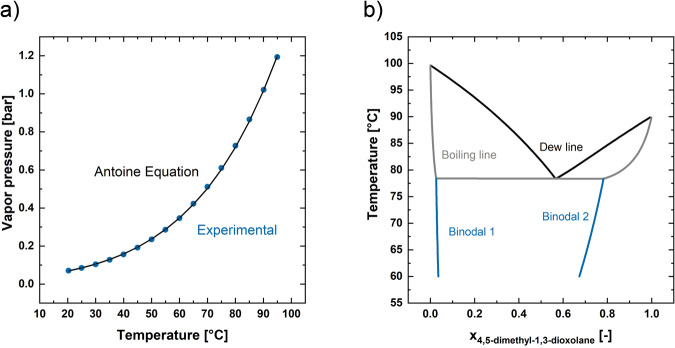
Property data of 4,5-dimethyl-1,3-dioxolane. Vapor pressure data of 4,5-dimethyl-1,3-dioxolane (**a**) and VLLE of 4,5-dimethyl-1,3-dioxolane and water (**b**) derived from COSMO-RS predictions.

To model the phase separation of 4,5-dimethyl-1,3-dioxolane and water as well as the consecutive distillation, vapor-liquid-liquid equilibrium (VLLE) data are necessary. The VLLE of the novel compound 4,5-dimethyl-1,3-dioxolane was predicted using COSMO-RS. To reduce the uncertainty of the COSMO-RS VLLE prediction, experimental vapor pressure data of 4,5-dimethyl-1,3-dioxolane was integrated into the calculations. Experimental vapor pressure data was obtained using the RAMSPEQU setup as described in ref. ^39^. Before each measurement in the RAMSPEQU setup, the equilibrium cell was thoroughly cleaned with ethanol as a cleaning agent. To remove the solvent residues after cleaning, the equilibrium cell was evacuated by a vacuum pump. To avoid contamination on opening the equilibrium cell, e.g., by atmospheric humidity, the cell was filled with nitrogen at a slight overpressure to the surrounding atmosphere. Subsequently, the equilibrium cell was filled with pure liquid substance through a syringe by removing the Pt100 from its holder. The Pt100 is then reattached to close the equilibrium cell. The pure liquid substance is degassed in situ by repeatedly opening the valve to vacuum. The degassing was completed when the pressure value remains the same after two successive degassing cycles. After degassing, the vapor pressure of the pure substance was measured. For the vapor pressure measurement, the desired temperature was set via a thermostat. Equilibrium is assumed to be reached as soon as the deviation of the temperature measured in the cell is within 0.1 K and the deviation of the pressure is within 0.2 kPa for at least 5 min^39^. The corresponding vapor pressure data of pure 4,5-dimethyl-1,3-dioxolane is presented in Fig. 12a. When applying linear interpolation between the measured pressure levels, the boiling point of 4,5-dimethyl-1,3-dioxolane can be determined to 89.33 °C at 1 bar. Antoine parameters (A, B, C) of the form

$$\log \left({p}_{s}\right)=A-\frac{B}{T+C}$$

were derived from regression (*A* = 10.4172, *B* = 4813.9946, *C* = 209.2139), where ${p}_{s}$ is the vapor pressure in bar and $T$ the corresponding temperature in Kelvin. The derived Antoine parameters were applied for consecutive VLLE calculation in COSMO-RS. The resulting VLLE in presented in Fig. 12b. A heteroazeotrope is predicted for the binary mixture with a boiling temperature of approximately 77 °C. The broad miscibility gap facilitates the separation of 4,5-dimethyl-1,3-dioxolane from water. A water rich phase and an phase rich in cyclic acetal can be obtained via decantation at mild temperature, which can be further purified via distillation. To use the obtained property data for process design, binary NRTL parameters were derived in the AspenPlus regression mode.

#### Process modeling in AspenPlus

AspenPlus V11.0 was utilized for the modeling of the process concepts. To cover non-idealities of the liquid mixtures an activity coefficient model is desired. The NRTL property method was chosen, since it is suitable for polar mixtures far away from the critical region at low pressure^40^. To model the extraction of acetoin and 2,3-BDO based on the ternary LLE data obtained from COSMO-RS, the NRTL parameters of the ternary systems were fitted to the LLE data by using the AspenPlus internal regression mode (see Table S1 and Table S2 in the Supplementary Methods in Section 1.3 Process Modeling). The NRTL parameters for the novel 4,5-dimethyl-1,3-dioxolane were determined analogously by fitting the binary interaction parameters to VLLE data (see Table S3 in Supplementary Methods in Section 1.3 Process Modeling).

The fermenter for the production of acetaldehyde from glucose was modeled at 30 °C and 1 bar. The yield of the conversion to acetaldehyde was set to 0.4655 g/g_glucose_, which equals to 95% of the theoretical yield (0.49 g/g_glucose_), while the remaining glucose is converted to CO_2_. A feed of $10{kg}/h$ glucose was applied, leading to an aeration of 150 L/min, assuming a productivity (*P*) and volumetric flow rate (*vvm*) as described previously. The absorption column is modeled by using the radfrac model with 5 theoretical separation stages, as discussed previously. The options of the condenser and reboiler are set to “None” and the gaseous feed is applied on stage 5, while the solvent is applied above stage 1. The pressure was set to 1 bar. The enzymatic cascade was modeled by using two sequential stoichiometric reactors at 30 °C and 1 bar, to describe the ligation and reduction step individually. The yield of the ligation was set to 0.8 in aqueous solvent and to 0.7 in MARS for the conversion of acetaldehyde to acetoin, analogous to the yield observed in the experiments. Thereby, the acetaldehyde concentration, which is fed to the reactor, is constraint to a maximum of 200 mM in order to avoid enzyme inactivation due to high aldehyde concentration. The reduction is modeled with a yield of 0.7 in aqueous solvent and 0.6 in MARS for the conversion of acetoin to 2,3-BDO. A yield of 1.0 is assumed for the conversion of acetaldehyde to ethanol, so that unreacted acetaldehyde cannot be recycled, since it is reduced to ethanol by the ADH. Both reactions in the second reactor need an electron donor for the co-factor recycling. Therefore, isopropanol is added in equimolar amount and is oxidized to acetone by the oxidoreductase. The extraction is modeled by utilizing the multistage extraction column model with a number of theoretical separation stages of 7 for the extraction of acetoin and five stages for 2,3-BDO at 25 °C. The chemical reaction of 2,3-BDO to 4,5-dimethyl-1,3-dioxolane is modeled by using a stoichiometric reactor at 90 °C and 80 bar. CO_2_ and H_2_ are applied in a molar ratio of 1:3 and CO_2_ is added in equimolar ratio to the amount of 2,3-BDO in the reactor. The remaining hydrogen is separated with a flash after the reactor at 1 bar and 30 °C. The chemical reactor for the conversion of acetoin to 2,3-BDO is modeled identical, but only H_2_ is applied at 100 bar and 90 °C for the hydration step of acetoin. Full conversion of the chemical reaction steps is assumed. Analogous to the method presented by Spöring et al., all distillation columns were designed by setting the distillate to feed ratio via design specs to achieve the desired purity of 99 w% and consecutively reducing the reflux ratio to reduce the necessary energy demand. Details regarding this procedure can be found in the Supplementary Methods in Section 1.3 Process Modeling, where the proceeding is presented exemplary for the benchmark process (Figs. S6, S7, and S8)^19^.

The assessment of the process concepts was conducted via specific energy demand and the overall yield, including the yields for conversion and separation. The specific energy demand is calculated via the heat duty $\dot{Q}$ for the reactors, distillation columns and cooling units and is normalized by the mass flow ${\dot{m}}_{{{{{{\rm{product}}}}}}}$ of the product (4,5-dimethyl-1,3-dioxolane).

$${specific\; energy\; demand}=\frac{\sum \dot{Q}}{{\dot{m}}_{{{{{{\rm{product}}}}}}}}$$

To retain the utilized whole cells within the biotechnological reactors, ultrafiltration can be applied, which is typically not intensive in terms of energy and is therefore neglected^13^.

### Process analysis

The described process routes and the permutations of possible downstream concepts for each route were evaluated in terms of specific energy demand and overall yield. The two routes (**R1** and **R2**) with each of three downstream concepts (distillation from aqueous solvent: **DIST-AQ**, distillation from MARS: **DIST-ORG**, extraction into aqueous solvent: **EXT-DIST**) for the separation of the enzymatically synthesized products (acetoin, 2,3-BDO) lead to six different concepts in total. Thereby, the first step is always the production of acetaldehyde and consecutive separation via absorption (see Fig. 6). Depending on the process route the intermediates 2,3-BDO or acetoin are formed and separated via distillation from aqueous solvent (**DIST-AQ**), distillation from MARS (**DIST-ORG**), or extraction into aqueous solvent (**EXT-DIST**) as presented in Fig. 7. The last process step is the acetalization and separation via distillation as presented in Fig. 9.

**Fig. 13:**
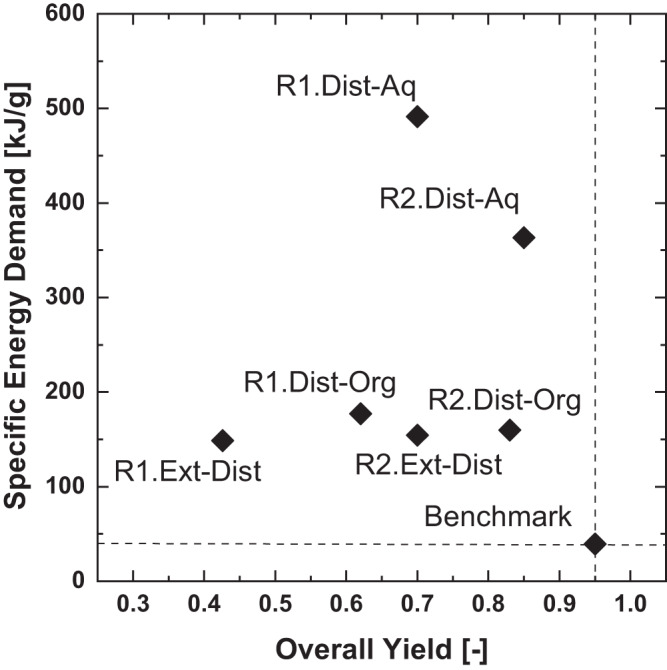
Assessment of the permutations of the two process routes. Assessment of the permutations of the two process routes (**R1,** **R2**) and the downstream concepts (**DIST-AQ,** **DIST-ORG,** **EXT-DIST**) for the enzymatically synthesized products (acetoin, 2,3-BDO). The process concepts were evaluated via specific energy demand and overall yield in a Pareto plot, including the benchmark process as reference. **R2.DIST-ORG** is the most favorable concept of the permutation due to the highest overall yield and the lowest specific energy demand, but still is less efficient the the benchmark process.

The resulting six process concepts (see Fig. 8) were assessed using the achieved yields from the wetlab experiments as described in Section 2. The specific energy demand and the corresponding overall yield of these six routes and the benchmark process are presented in Fig. 13 in a Pareto plot.

The process concepts that are based on the first route (**R1**) show a lower overall yield than the concepts based on the second route (**R2**). This is due to the full conversion of acetoin to 2,3-BDO in the chemo-catalytic hydration (**R2**), while the enzymatic reduction step only has a yield of 0.7 for aqueous solvent and a yield of 0.6 for MARS (**R1**). The specific energy demand of the distillation of 2,3-BDO from water (**DIST-AQ**) is the highest for both routes (**R1** and **R2**), since the evaporation of large amounts of water to obtain the diluted enzymatically produced compounds (acetoin or 2,3-BDO) is unfavorable. The distillation from MARS (**DIST-ORG**) has a significantly reduced specific energy demand compared to **DIST-AQ** for both routes since only the organic reaction solvent (CPME) needs to be evaporated. However, the overall yield of **DIST-ORG** is slightly reduced, since the enzymatically conversion in the organic reaction solvent has a lower yield than the conversion in aqueous phase. The extraction-based concept (**EXT-DIST**) is the most efficient in terms of specific energy demand for both process routes (**R1** & **R2**) while its overall yield is the lowest due to losses in extraction as an additional separation step. The most favorable combination of reaction route and downstream concept is the second route (**R2**) with chemical conversion of acetoin, which was synthesized enzymatically in MARS. The contribution of each conversion and corresponding separation step to the overall specific energy demand is analyzed for the best overall process concept (**R2.DIST-ORG**) besides the benchmark process. The fraction of the specific energy demand accounted for the fermentative production, and the downstream processing of acetaldehyde corresponds to 13.5% of the total demand. The largest fraction is made up of the production and purification of the diluted enzymatically produced compounds (acetoin and 2,3-BDO) with 82.2 %. The acetalization and separation of the gained product only accounts for the remaining 4.3%, since the 4,5-dimethyl-1,3-dioxolane is formed in high concentration. Therefore, its purification is less energy intensive compared to separation of the diluted biotechnological products.

**Fig. 14:**
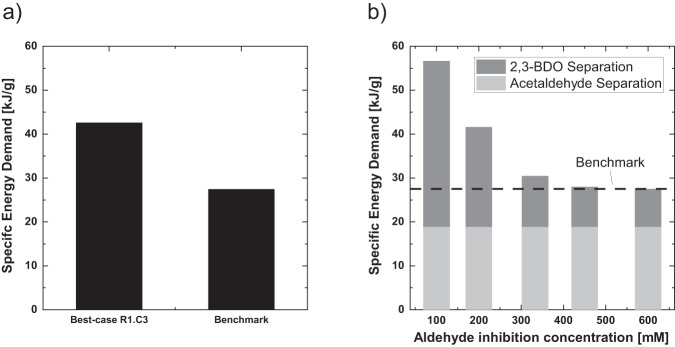
Process assessment. Specific energy demand for the best-case process in comparison to the direct fermentation to 2,3-BDO (reference process). The reference process is more efficient in terms of specific energy demand (**a**). The maximal applicable acetaldehyde concentration, which is limited due to enzyme stability, is varied to assess the sensitivity of the process to this performance parameter (**b**). A break-even point with the reference process is found at 600 mM.

In the following, the best overall process concept developed in this work is compared to the benchmark process, which is more efficient due to its high titers and yields. The fermentative production of 2,3-BDO and its downstream was extensively investigated in literature^9,10,13^. Harvianto *et al*. calculated the specific energy demand (reboiler duty) for the separation of 2,3-BDO from water to 24.74 kJ/g as their base case^9^. Haider *et al*. found a specific energy demand for the separation of 2,3-BDO from its aqueous fermentation medium of 28.68 kJ/g^10^. We calculated a specific energy demand for separation of 2,3-BDO from water of 27.5 kJ/g. Hence, the mean value of these three studies is defined as benchmark (26.97 kJ/g). To assess the potential of the developed routes consisting of the novel conversion steps mentioned is this work in a fair way, best-case scenarios are created, since the novel conversion steps are not as far developed as the 2,3-BDO fermentation from the benchmark process. Hence, the yields of the enzymatic conversion steps were set to full conversion. Full conversion of an extensively developed and optimized enzymatic cascade appears to be reasonable, since previous studies achieved yields close to unity in comparable cascades, even in the MARS system^18^. Additionally, the amount of used solvent for the absorptive and extractive separation sequences was set to a minimum and the number of separation stages was increased. These assumptions lead to a reduced specific energy demand compared to the assessment presented in Fig. 13. Since both possible conversion steps for the production of 2,3-BDO from acetoin now have a yield of 1.0, the chemo-catalytic hydration conversion is no longer advantageous compared to the enzymatic reduction. The separation of 2,3-BDO via extraction, however, is more efficient than the separation of acetoin via distillation. Hence, the most efficient process concept is **R1.EXT-DIST**, when considering the best-case process. The optimizations of the process conditions for the best-case processes led to a specific energy demand of 42.33 kJ/g (see Fig. 14a).

It is evident that the separation of 2,3-BDO in the reference process is more efficient in terms of specific energy demand. The specific energy demand of the developed best-case process needs to be reduced by 32.2% to be competitive. As mentioned above, the largest fraction of the energy demand is referred to the purification of the enzymatically produced compounds acetoin or 2,3-BDO, due to the highly diluted product solution. The low concentrations of the target products mainly result from the low enzyme stability against acetaldehyde. This leads to a maximum feed concentration of acetaldehyde of 200 mM. Since this performance parameter is crucial for the overall specific energy demand of the process and more stable enzymes can be developed potentially using e.g., enzyme engineering, a sensitivity analysis is performed to assess the impact of enhanced enzyme stability. In Fig. 14b, the maximum aldehyde concentration is varied and the resulting specific energy demand for the overall process towards 2,3-BDO is calculated. The specific energy demand is distinguished between specific energy demand for the separation of acetaldehyde from the fermentation and specific energy demand for separation of 2,3-BDO. Due to a higher possible concentration of the acetaldehyde in the enzymatic cascade, a higher product concentration is achieved, and the separation is more efficient. When assuming a possible acetaldehyde concentration of 600 mM, a break-even point with the reference process was found. Therefore, the enzyme stability was identified as a major performance parameter in combination with the yields of the individual conversion steps.

## Conclusion

Novel process routes consisting of multiple reaction steps to produce the promising bio-hybrid fuel 4,5-dimethyl-1,3-dioxolane from glucose were developed. The precursor for the acetalization is 2,3-butanediol, which is an intermediate product in all process routes. The individual reaction steps of the glucose-based synthesis of 4,5-dimethyl-1,3-dioxolane are performed with microbial, enzymatic, and chemical catalysts in scenarios with varying combinations. A proof-of-concept for each conversion step was established. To link these production steps and to meet the constraints of the individual catalysts, suitable separation techniques were identified. When linking the individual conversion steps to possible process routes, degrees of freedom are the selected catalysts (microbes, enzymes, chemo-catalyst) for each conversion step as well as the reaction solvent. The resulting process concepts were implemented in AspenPlus and assessed via product specific energy demand as well as overall yield. The most efficient route to produce 4,5-dimethyl-1,3-dioxolane was identified and the bottleneck was found to be the enzymatic conversion. Thereby, the limiting performance parameter is the stability of the enzymes with respect to high acetaldehyde concentrations, resulting in low concentrated educt and product streams. A best-case scenario for the developed process variations was compared to a reference process to produce 2,3-butanediol from glucose taken from literature. A target setting was performed for the enzyme stability and hence, the catalyst design. To be competitive with the reference process in terms of specific energy demand, the enzymatic stability needs to be improved to an aldehyde concentration of 600 mM to achieve increased product concentrations. This can be targeted in the future by making use of enzyme engineering (rational or random, e.g., via directed evolution, or a combination thereof) as well as catalyst protection via immobilization of the enzymes. Further, fed-batch reaction mode might lead to enhanced product concentration, however, decreased productivity. The here presented approach of combining different catalysts and solvent systems with model evaluation is promising to exploit the ever-growing toolbox of catalysis, for carbon-efficient synthesis of interesting molecules.

## Methods

### Enzymatic catalysis

See Supplementary Section 1 and Supplementary Figs. S1, S2.

### Chemocatalytic conversion

See Supplementary Section 2 and Supplementary Figs. S3, S4.

### Process modeling

See Supplementary Section 3, Supplementary Tables S1–S3, and Supplementary Figs. S5–S8.

### Supplementary information

Supplementary Information

## Data Availability

All data generated during the current study are available from the corresponding author on reasonable request.
