# Author Correction: Interdisciplinary development of an overall process concept from glucose to 4,5-dimethyl-1,3-dioxolane via 2,3-butanediol

**DOI:** 10.1038/s42004-023-01089-9

**Published:** 2023-12-21

**Authors:** William Graf von Westarp, Jan Wiesenthal, Jan-Dirk Spöring, Hendrik G. Mengers, Marvin Kasterke, Hans-Jürgen Koß, Lars M. Blank, Dörte Rother, Jürgen Klankermayer, Andreas Jupke

**Affiliations:** 1 Fluid Process Engineering (AVT.FVT), RWTH Aachen University, Forckenbeckstraße 51, 52074 Aachen, Germany; 2 Institute of Technical and Macromolecular Chemistry (ITMC), RWTH Aachen University, Worringerweg 2, 52074 Aachen, Germany; 3 Institute for Bio- and Geosciences Plant Sciences (IBG-1), Forschungszentrum Jülich GmbH, Wilhelm-Johnen-Straße, 52428 Jülich, Germany; 4 Aachen Biology and Biotechnology (ABBt), RWTH Aachen University, Worringerweg 3, 52074 Aachen, Germany; 5 Institute of Applied Microbiology (iAMB), Aachen Biology and Biotechnology (ABBt), RWTH Aachen University, Worringerweg 1, 52074 Aachen, Germany; 6 Institute of Technical Thermodynamics (LTT), RWTH Aachen University, Schinkelstraße 8, 52062 Aachen, Germany

**Keywords:** Chemical engineering, Biocatalysis, Sustainability, Process chemistry

Correction to: *Communications Chemistry* 10.1038/s42004-023-01052-8, published online 16 November 2023.

The original version of this Article and Supplementary Information contained errors with respect to the designation of the corresponding authors.

William Graf von Westarp was incorrectly designated as a corresponding author and his e-mail address was listed in error. Meanwhile, Jürgen Klankermayer and Andreas Jupke were erroneously designated as non-corresponding authors, and their e-mail addresses were omitted in error.

These errors have now been corrected in the PDF and HTML versions of the Article.

