# Supplementary material for: Interdisciplinary development of an overall process concept from glucose to 4,5-dimethyl-1,3-dioxolane via 2,3-butanediol
Source: Commun Chem. 2023 Nov 16;6:253. doi: 10.1038/s42004-023-01052-8 (PMC10654704; doi:10.1038/s42004-023-01052-8)
Supplement: Supplementary file 1 — Supplementary Information [file 42004_2023_1052_MOESM1_ESM.docx]

Interdisciplinary development of an overall process concept from glucose to 4,5-dimethyl-1,3-dioxolane via 2,3-butanediol

William Graf von Westarp^a^, Jan Wiesenthal^b^, Jan-Dirk Spöring^c,d^, Hendrik Mengers^e^, Marvin Kasterke^f^, Hans-Jürgen Koß^f^, Lars M. Blank^e^, Dörte Rother^c,d^, Jürgen Klankermayer^b^, Andreas Jupke^a^

1. Fluid Process Engineering (AVT.FVT), RWTH Aachen University

b. Institute of Technical and Macromolecular Chemistry (ITMC), RWTH Aachen University

c. Institute for Bio- and Geosciences Plant Sciences (IBG-1), Forschungszentrum Jülich

d. Aachen Biology and Biotechnology (ABBt), RWTH Aachen University

e. Institute of Applied Microbiology (iAMB), RWTH Aachen University

f. Chair of Technical Thermodynamics (LTT), RWTH Aachen University

**Corresponding author**: Andreas Jupke, Jürgen Klankermayer

**E-mails:** [JKlankermayer@itmc.rwth-aachen.de](mailto:JKlankermayer@itmc.rwth-aachen.de), [andreas.jupke@avt.rwth-aachen.de](mailto:andreas.jupke@avt.rwth-aachen.de)

**Keywords**: 4,5-dimethyl-1,3-dioxolane, 2,3-butanediol, acetaldehyde, process design, molecular catalysis, sustainable synthesis

1. **Enzymatic catalysis**

In the following section, the details regarding the enzymatic conversion steps are described.

- 1. Catalyst production

For the production of the catalyst formulated as lyophilized whole cells, the following protocol was used. The benzaldehyde lyase from *Pseudomonas fluorescens* (*Pf*BAL) (GenBank AY007242.1) and alcohol dehydrogenase from *Lactobacillus brevis* (*Lb*ADH) (GenBank CAD66648.1), were produced using *Escherichia coli* BL21(DE3) in autoinduction medium at 37 °C for 2 hours followed by 48 h incubation at 20 °C with 90 rpm [28]. The cell suspension was centrifuged for 20 minutes at 4000 *x* *g* at 4 °C. The resulting cell pellet was subsequently frozen at -20 °C before it was lyophilized at -54 °C and 0.01 mbar using a Christ Alpha 1-4 LD plus freeze drier (Martin Christ Gefriertrocknungsanlagen, Osterode am Harz, Germany). The dried pellets were crushed using a mortar and stored at -20 °C.

- 1. Conduction

For the examination of the described reaction in MARS, 30 mg of LWC as catalyst were weighed into a 1.5 mL glass vial and CPME and 200 mM of aldehyde was added to a final volume of 1 mL. To start the reaction, 30 µl of 1 M TEA buffer pH = 9 with 2.5 mM MgCl_2_ and 0.1 mM thiamine diphosphate was added and the mixture was stirred at 1000 rpm at 30°C. After 120 min the *Pf*BAL was removed by centrifugation (3 min, 14,000 rpm) and the supernatant was added to 30 mg of *Lb*ADH LWC. The reduction reaction was started by adding 30 µl of 1 M TEA buffer pH = 9 with 2.5 mM MgCl_2_ and 0.1 mM thiamine diphosphate. For cofactor regeneration of the reduction step, 1 M of isopropanol was added.

For the investigation of the enzymatic conversion in aqueous buffered solvent, an identical setup as of the enzymatic reactions in MARS was prepared. Instead of CPME, 50 mM triethanolamine buffer pH = 9 with 2.5 mM MgCl_2_ and 0.1 mM thiamine diphosphate was used and no additional 1 M TEA was added to start the reaction.

- 1. Analytics via Gas Chromatography


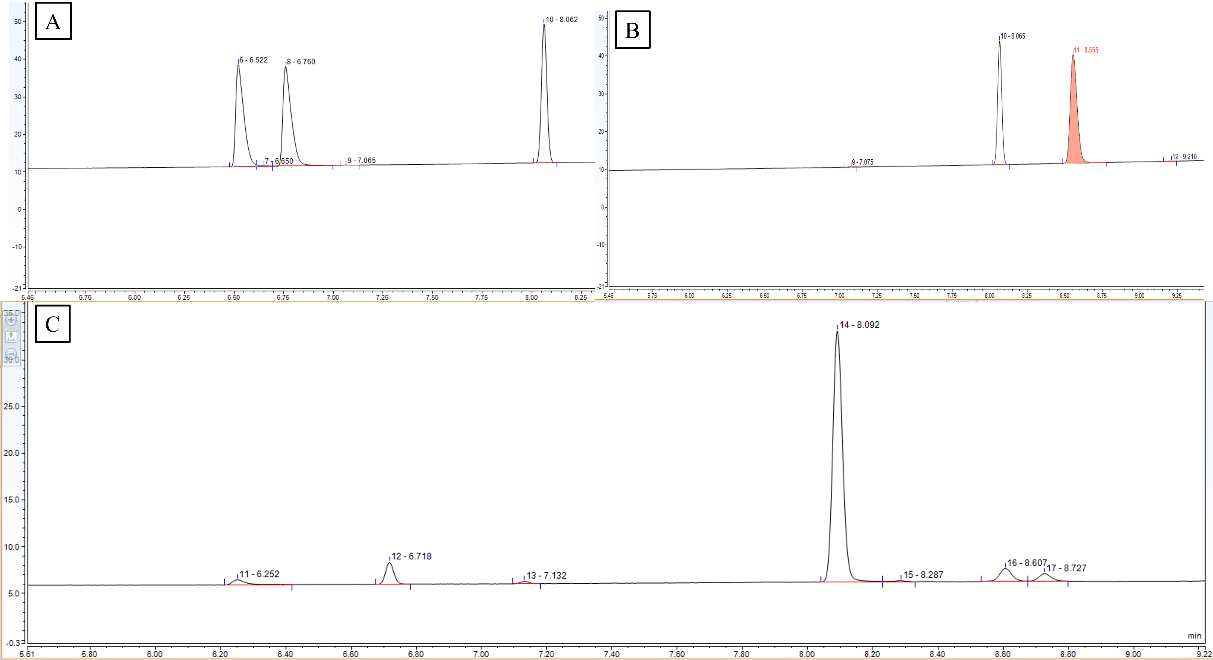
Sample volumes of 20 µL were drawn and diluted in 380 µL acetonitrile containing decane as internal standard. The samples were then centrifuged using an Eppendorf 5424 R centrifuge (Eppendorf, Hamburg. Germany) at 13,000 rpm (15,871 *x g*) to remove the whole cell catalyst and the supernatant was transferred to Thermo Fisher Trace 1310 GC (Thermo Fisher, Waltham, USA) vials for analysis. A CP Chirasil-Dex-CB 25 m x 0.25 mm x 0.25 µm column was used with an injection volume of 1 µL and an injector temperature of 250°C. A split of 50:1 was applied using a temperature gradient (40°C for 0.5 min, 17°C/min until 55°C for 0.5 min, 10°C/min until 73 °C then 15°C/min until 82 °C for 2.5 min, 20 °C/min until 117 °C for 2.2 min, 26 °C/min until 140 °C then 50 °C/min until 190 °C. Full runtime 16 min) Retention times: (*S*) Acetoin 6.76 min, (*R*) Acetoin 6.522 min, (2*S*,3*S*)-butanediol 8.453 min, (2*R*,3*R*)-butanediol 8.502 min, *meso‑*butanediol 8.735 (see Fig. 1).

**Figure S1:** GC Chromatograms of (A) racemic acetoin with peaks of (R)-acetoin at 6.522 min and (S)-acetoin at 6.759 min, (B) (2R,3R)-butanediol at 8.555 min, and (C) a reaction mixture with (S)-acetoin (6.78 min), (2R,3R)—butanediol (8.607 min) and meso-2,3-butanediol (8.727 min). All samples contain decane as an internal standard, visible at 8.09 min.

- 1. Results

In both reaction solvents the product formation was successful. The final concentration of 2,3-BDO in aqueous solvent (50 mM) is slightly higher compared to MARS (32 mM). Due to the simplified downstream processing from MARS, a trade-off between high product titer and downstream processing costs needs to found (see Figure S1).

*
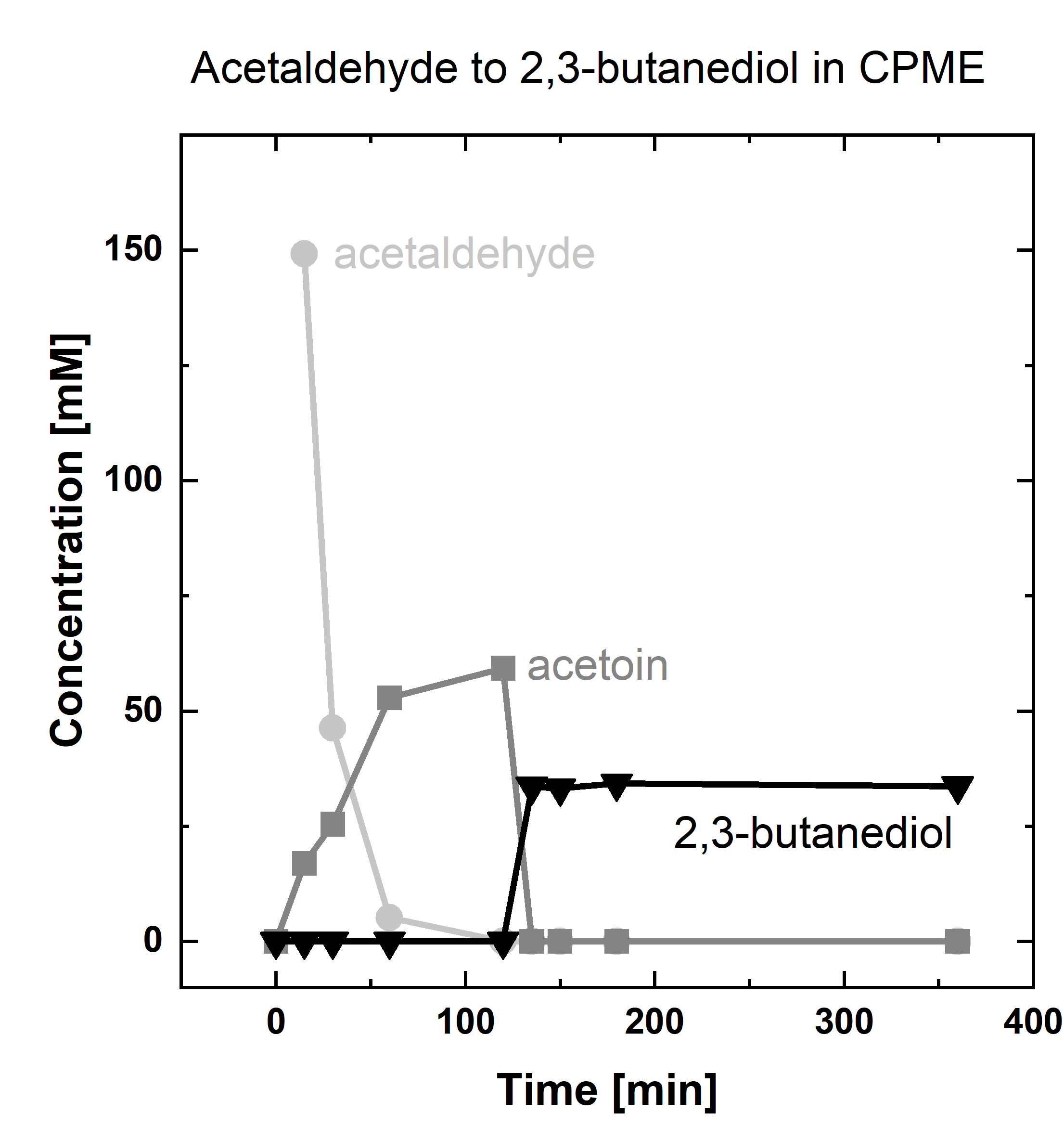

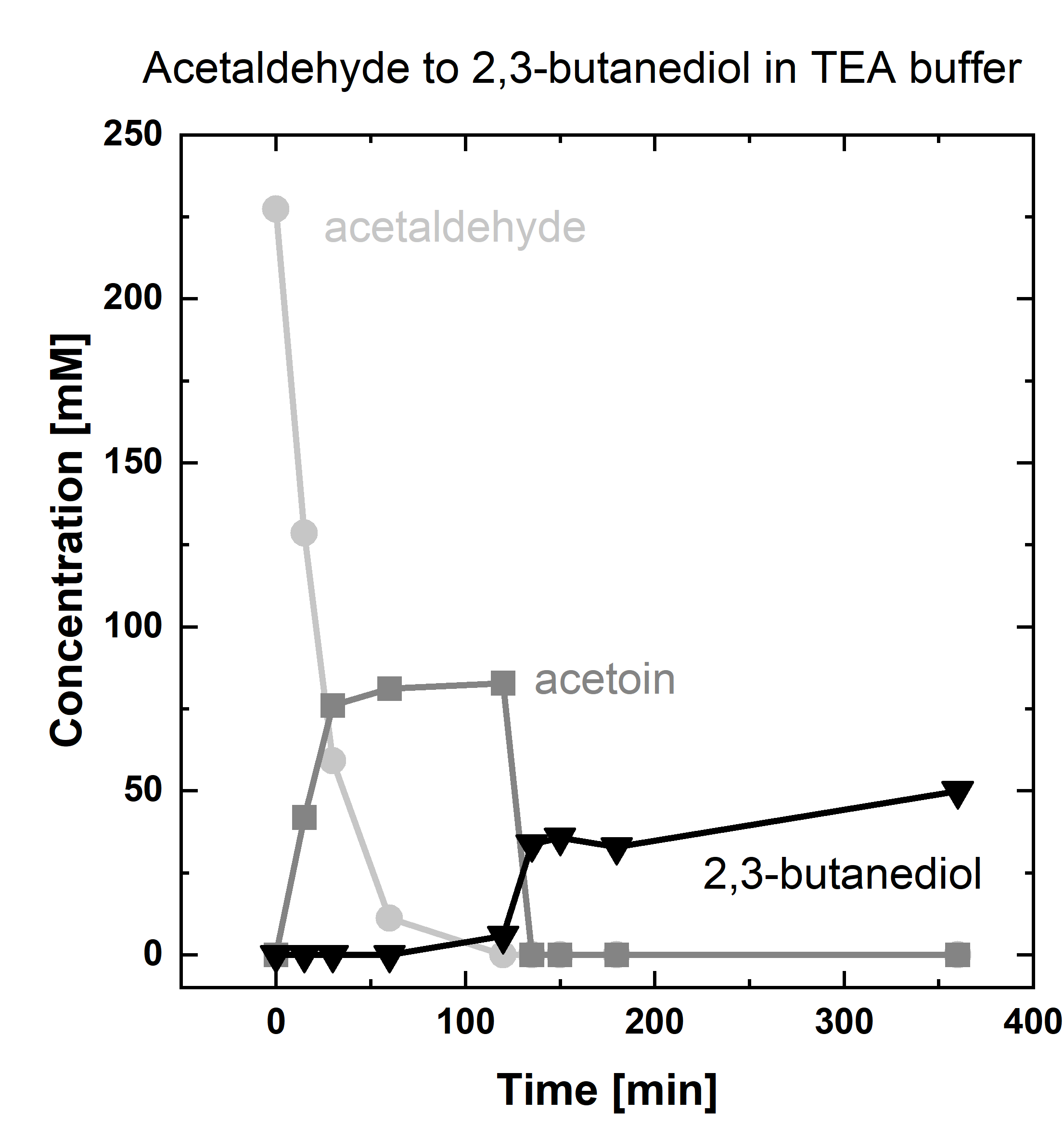
*

**Figure S2:** Two-step enzymatic conversion of acetaldehyde to 2,3-butanediol. In the first 120 min 200 mM acetaldehyde were ligated to acetoin. Subsequently, *Lb*ADH was added and the acetoin was reduced to 2,3-butanediol using 1 M isopropanol for cofactor regeneration. A: 2-step cascade under aqueous conditions with 50 mM TEA buffer as reaction solvent and B: under organic conditions with CPME as the reaction solvent. The concentrations of product were analysed via GC and the assays were performed as technical triplicates.

1. **Chemocatalytic Conversion**

All experiments were performed using an inert argon atmosphere following standard Schlenk techniques. Air or moisture-sensitive substances were stored using a Glovebox (*Innovative Technology* PL-4GB-1800-EM, *MBraun*, type MB150BG) or Schlenk flasks. Argon 4.8, procured from the company *Air Products* (99,998 %) and purified by a gas cleaning facility *MBraun* MB 100HP, was used as the inert gas.

- 1. Catalyst production

The used ruthenium based chemocatalyst was produced as presented in literature [32, 33]. It consists of 1,1,1-tris(diphenylphosphinomethyl)ethane (624.9 mg, 1.0 mmol, 1.0 eq) and and [Ru(COD)(methylallyl)_2_] (319.7 mg, 1.0 mmol, 1.0 eq), which are weighted into a 50 mL Schlenk tube. The substrates are dissolved in 15 mL mesitylene and stirred for 5 min at room temperature. In an oil bath, the reaction mixture is heated to 145 °C for 16 h. The solution turned black and a colorless solid precipitated. *n*‑pentane (10 mL) was added to enhance the product precipitation. The supernatant is removed with a filter cannula. The filter cake is washed with 3 · 5 mL *n*-pentane and dried *in vacuo* for 6 h. The product was obtained as an ochre-colored powder (440 mg, 55 %).

^1^H-NMR (400 MHz, 400 MHz, CDCl_3_): δ = 7.02-6.94 (m, 18H, C_Ar_-H), 6.89-6.82 (m, 12H, C_Ar_‑H), 2.15 (bs, 6H, P-CH_2_), 1.57 (bs, 6H, C-CH_2_), 1.31 (s, 3H, CH_3_) ppm.

^31^P-NMR (162 MHz, CDCl_3_): δ =34.8 (s, 3P) ppm.

- 1. Conduction

To produce 4,5-dimethyl-1,3-dioxolane via acetalization CO_2_, 2,3-butanediol (54.5 mg, 0.6 mmol), [Ru(triphos)(tmm)] (6.21 mg, 0.008 mmol) and HNTf_2_ (6.2 mg, 0.022 mmol) were weighted into an inlet inside the glovebox. The inlet was placed inside a 20 mL finger autoclave and an inert gas atmosphere was established. In an argon counter-flow, CPME (2 mL) was added and the autoclave was sealed. At r.t., first, CO_2_ (20 bar) followed by H_2_ to a combined pressure of 100 bar was pressed onto the autoclave. In an aluminum cone, the autoclave was subsequently heated to 90 °C for 16 h. After the reaction time, the autoclave was cooled in an ice bath and carefully vented. The reaction mixture was analyzed using ^1^H-NMR and 13C-NMR spectroscopy with mesitylene as the internal standard. The cyclic acetals were obtained in 16 % yield.

For a one-pot, two-step reaction from hydroxy ketones to cyclic acetals using CO_2_, the 20 ml autoclave inlet was filled with [Ru(triphos)(tmm)] (3.88 mg, 0.005 mmol) and HNTf_2_ (1.68 mg, 0.0060 mmol) inside the glovebox. The inlet was placed inside the autoclave and an inert gas atmosphere was established. Acetoin (50.7 mg, 0.575 mmol) and CPME (2 mL) was added, and the autoclave was pressurized with H_2_ (100 bar). After the reaction time of 16 h at 120 °C, the autoclave was cooled in an ice bath and vented to 10 bar. The autoclave was loosely attached to a Schlenk line and the remaining pressure was carefully released with an argon counter current. After attaching the autoclave tightly to the Schlenk line, it was opened and [Ru(triphos)(tmm)] (2.31 mg, 0.003 mmol) and HNTf_2_ (4.69 mg, 0.0167 mmol) solved in CPME (1 mL) was added. The autoclave was sealed and CO_2_ (20 bar) and H_2_ to a combined pressure of 100 bar was added. In an aluminum cone, the autoclave was heated to 90 °C for 16 h. After the reaction time, the autoclave was cooled in an ice bath and carefully vented. The reaction mixture was analyzed with ^1^H-NMR spectroscopy using mesitylene as an internal standard. The cyclic acetals were obtained in 18 % yield.

- 1. Analytics

Nuclear magnetic resonance (NMR) spectra of the diastereomeric mixture of 4,5-dimethyl-1,3-dioxolanes were measured at r.t. with *Bruker* *AV-300* (^1^H: 299.6 MHz, ^13^C: 75.3 MHz, ^31^P: 121.3 MHz) and *Bruker AV-400* (^1^H 400.2 MHz, ^13^C: 100.6 MHz, ^31^P: 162.0 MHz), *Bruker AS-400* (^1^H: 400.2 MHz, ^13^C: 100.6 MHz, ^31^P: 162.0 MHz) spectrometers (see Fig. S3 and Fig. S4). The chemical shift was determined in ppm, and the spectra were referenced relative to the residual proton signal of the deuterated solvents. The multiplicities were annotated as singlet, doublet, triplet or multiplet. Broad signals are indicated with “bs”. The coupling constants were displayed in Hertz.

**(4*S*,5*S*)-dimethyl-1,3-dioxolane**

^1^H NMR (400 MHz, CDCl_3_): δ_H_ = 4.88 (2H, s, O-CH2-O), 3.57-3.51 (2H, m, CH), 1.21-1.19 (6H, m, -CH_3_) ppm.

13C{1H} NMR (101 MHz, CDCl3) δ =93.78 (s, CH2), 78.52 (s, CH), 16.74 (CH3) ppm.

***meso*-4,5-dimethyl-1,3-dioxolane**

^1^H NMR (400 MHz, CDCl_3_): δ_H_ = 4.82 (2H, d, O-CH2-O), 4.05-4.01 (2H, m, CH), 1.08-1.06 (6H, m, -CH_3_) ppm

13C{1H} NMR (101 MHz, CDCl3) δ = 93.44 (s, CH2), 73.95 (s, CH), 14.57 (CH3) ppm.


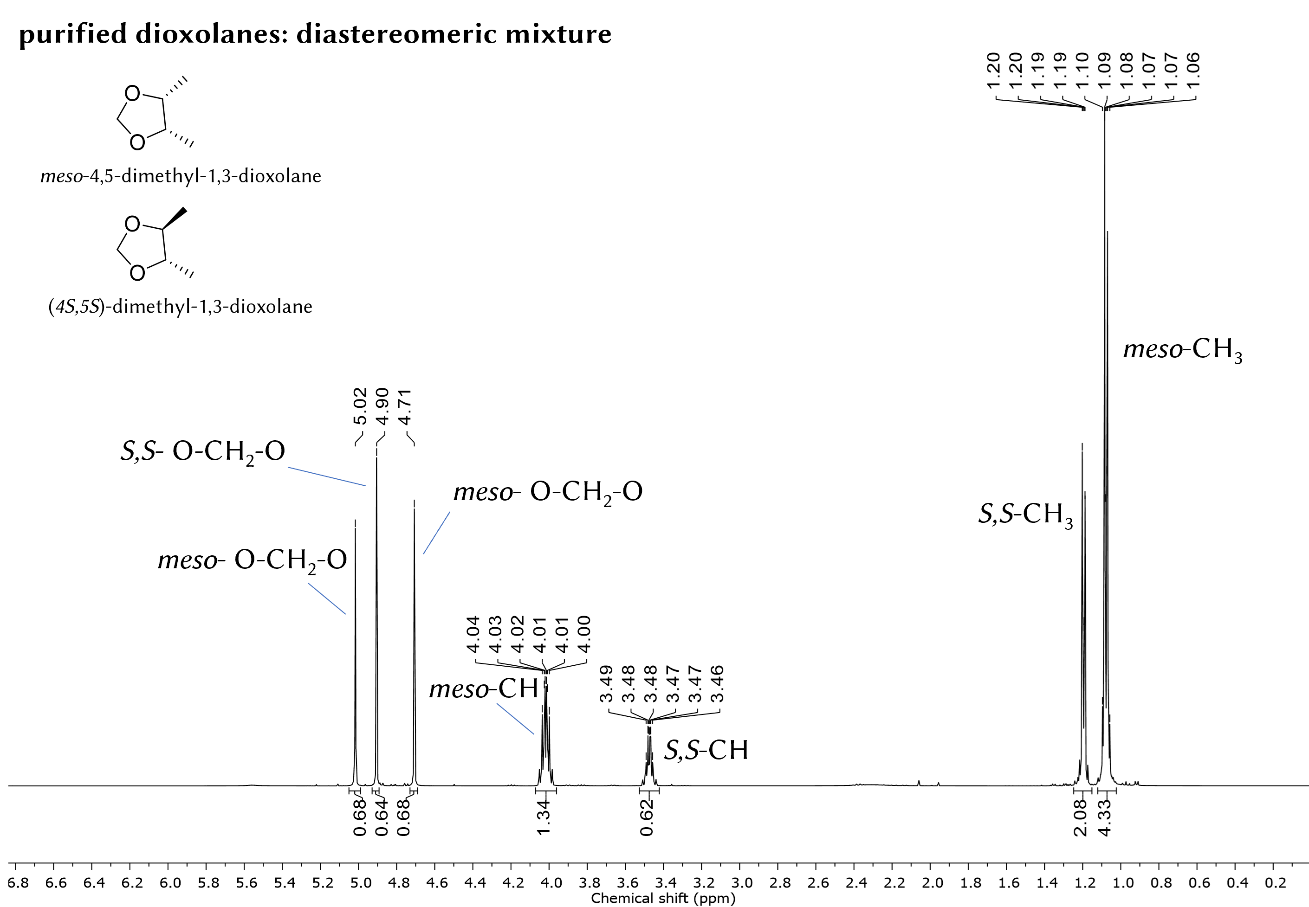


**Figure S3:** ^1^H NMR (400 MHz, CDCl_3_) spectrum of a diastereomeric mixture 4,5-dimethyl-1,3-dioxolanes.

**Figure S4:** ^13^C{^1^H} NMR (101 MHz, CDCl_3_) spectrum of a diastereomeric mixture of 4,5-dimethyl-1,3-dioxolanes.


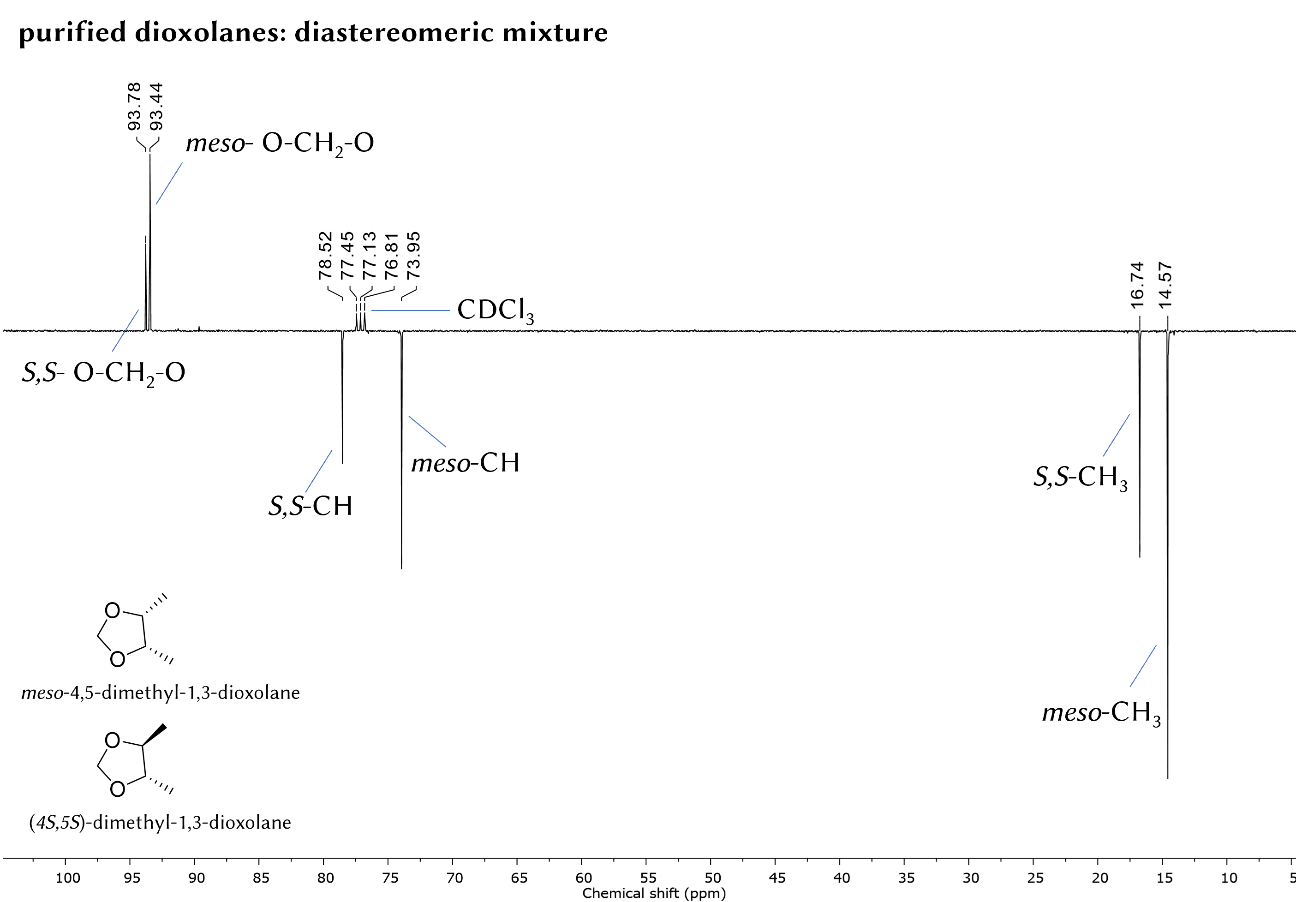


1. **Process Modelling**

In the following, details regarding the used parametrization of the NRTL model as well as proceedings of the process modelling can be found.

- 1. Parametrization

**Table 1**: Binary interaction NRTL parameters for Extraction of 2,3-BDO from CPME. Residual root mean square error = 16.42 when fitting to COSMO-RS LLE data.

| Compound | Aij | Aji | Bij | Bji | Cij |
| --- | --- | --- | --- | --- | --- |
| H2O:2,3-BDO | -0.831 | -0.566 | 538.888 | -88.43 | 0.3 |
| H2O:CPME | 2.43 | 30.617 | 814.701 | -8080.561 | 0.3 |
| 2,3-BDO:CPME | -26.445 | 31.853 | 8078.714 | -9002.5 | 0.3 |

**Table 2**: Binary interaction NRTL parameters for Extraction of acetoin from CPME. Residual root mean square error = 989.89 when fitting to COSMO-RS LLE data.

| Compound | Aij | Aji | Bij | Bji | Cij |
| --- | --- | --- | --- | --- | --- |
| CPME:H2O | -10.203 | -6.78 | 3598.3 | 3384.32 | 0.235 |
| Acetoin:H2O | -2.845 | -4.964 | -252.24 | 790.12 | 0.3 |
| Acetoin:CPME | 2.461 | -3.727 | 74.5 | 615.2 | 0.3 |

**Table 3**: Binary interaction NRTL parameters for Phase separation and distillation of 4,5-dimethyl-1,3-dioxolane (Acetal) and water. Residual root mean square error = 7.16 when fitting to COSMO-RS VLLE data.

| Compound | Aij | Aji | Bij | Bji | Cij |
| --- | --- | --- | --- | --- | --- |
| Acetal:H2O | 7.999 | 3.988 | -2479.835 | -276.179 | 0.3 |

- 1. Proceedings in Process Modelling

For the process evaluation, the unit operation the conceptional processes presented were not optimized rigorously, rather sensitivity analysis of the crucial control variables was conducted.

As described in the manuscript, the amount of necessary solvent for extraction of acetoin or 2,3-BDO and the number of theoretical separation stages were investigated via sensitivity analysis in AspenPlus (see Figure S5).

**Figure S5:** For the extraction of acetoin (black squares) and 2,3-BDO (grey circles) the recovery was constraint to 95%. The resulting minimal amounts of solvent for varying number of theoretical separation stages can be found in Figure S5. For the case of acetoin, 7 theoretical separation stages and a corresponding solvent to feed ratio of 0.14 were chosen as a trade of between solvent demand and feasible height of the extraction column. For the extraction of 2,3-BDO, 5 theoretical separation stages and a solvent to feed ratio of 0.035 was chosen.

The design of the distillation columns in AspenPlus was performed as follows. The distillation column for the separation of 2,3-BDO from water in the benchmark process is chosen to presented the proceeding). The feed of the column consists of 10 w% 2,3-BDO in water. Firstly, a sensitivity analysis of the distillate to feed ratio was performed. To achieve the desired purity of 99 w% the distillate to feed ratio was increased with a fixed number of theoretical separation stages ($N_{th}=10$) and a fixed feed stage ($N_{feed}=$5). As presented in Figure S6, the achieved purity increases linearly with increased distillate to feed ratio. The distillate to feed ratio was found to be 0.97725 to separate 2,3-BDO and water and achieve a purity of 99.15 w% in the distillate.

**Figure S6:** For the design of the distillation columns, the necessary distillate to feed ratio to achieve a purity of 99 w% is determined with a reflux ratio of 0.1 kmol/h.

Consecutively, the reflux ratio is minimized while fullfilling the purity constraint using a distillate to feed ratio of 0.97725. The purity in the distillate is presented with respect to the reflux ratio in Figure S7.

**Figure S7:** For the design of the distillation columns, the necessary reflux ratio to achieve a purity of 99 w% is determined. When a distillate to feed ratio of 0.97725 is used, a reflux ratio of 0.04 kmol/h is sufficient to successfully separate 2,3-BDO from water.

Lastly, the reboiler duty with respect to the purity of 2,3-BDO in the distillate is shown in Figure S8. The reboiler duty is very sensitive to the settings of distillate to feed ratio and reflux ratio leading to the desired purity. Hence, the demonstrated procedure was conducted for alle distillation columns in this study to circumvent significant overestimation of the necessary specific energy demand.

**Figure S8:** The reboiler duty for the separation of 2,3-BDO from water is presented with respect to the desired purity. The reboiler duty increases with higher purities, demonstrating the necessity of a consistent procedure for the distillation column design.
